# A Statistical Design for Testing Transgenerational Genomic Imprinting in Natural Human Populations

**DOI:** 10.1371/journal.pone.0016858

**Published:** 2011-02-25

**Authors:** Yao Li, Yunqian Guo, Jianxin Wang, Wei Hou, Myron N. Chang, Duanping Liao, Rongling Wu

**Affiliations:** 1 Center for Computational Biology, Beijing Forestry University, Beijing, People's Republic of China; 2 Department of Statistics, West Virginia University, Morgantown, West Virginia, United States of America; 3 Department of Biostatistics, University of Florida, Gainesville, Florida, United States of America; 4 Department of Public Health Sciences, Penn State College of Medicine, Hershey, Pennsylvania, United States of America; Dana-Farber Cancer Institute, United States of America

## Abstract

Genomic imprinting is a phenomenon in which the same allele is expressed differently, depending on its parental origin. Such a phenomenon, also called the parent-of-origin effect, has been recognized to play a pivotal role in embryological development and pathogenesis in many species. Here we propose a statistical design for detecting imprinted loci that control quantitative traits based on a random set of three-generation families from a natural population in humans. This design provides a pathway for characterizing the effects of imprinted genes on a complex trait or disease at different generations and testing transgenerational changes of imprinted effects. The design is integrated with population and cytogenetic principles of gene segregation and transmission from a previous generation to next. The implementation of the EM algorithm within the design framework leads to the estimation of genetic parameters that define imprinted effects. A simulation study is used to investigate the statistical properties of the model and validate its utilization. This new design, coupled with increasingly used genome-wide association studies, should have an immediate implication for studying the genetic architecture of complex traits in humans.

## Introduction

Genomic imprinting arises from a gene when either the maternally or paternally derived copy of it is expressed while the other copy is silenced [1], [2]. Caused by epigenetic modifications such as DNA methylation established during gametogenesis and maintained throughout somatic development in the offspring, genetic imprinting has been shown to play a pivotal role in regulating the formation, development, function, and evolution of complex traits and diseases [3], [4], [5], [6], [7], [8], [9], [10]. While most studies of genetic imprinting focus on the epigenetic and molecular mechanisms of this phenomenon [7], [11], the number and distribution of imprinted genes and their epistatic interactions for quantitative traits are poorly understood, limiting the scope of our inference about the effects of imprinting genes on the diversity of biological traits or processes. Several authors have started to use genome-wide association and linkage studies to identify the regions of the genome that contain imprinted sequence variants and further understand the epigenetic variation of complex traits [12], [13], [14], [15].

In a series of recent studies, Cheverud, Wolf, and colleagues categorized genetic imprinting into different types based on the pattern of its expression, i.e., maternal expression, paternal expression, bipolar dominance, polar overdominance, and polar underdominance [14], [15]. With a three-generation F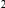 design, they identified these types of imprinted quantitative trait loci (iQTL) affecting body weight and growth in mice, displaying much more complex and diverse effect patterns than previously assumed. A different design based on reciprocal backcrosses was proposed to test and estimate the distribution of iQTL responsible for physiological traits related to endosperm development in maize [16]. By modeling identical-by-descent relationships in multiple related families of canines, Liu et al. [13] derived a random effect model based on linkage analysis to genome-wide scan for the existence of iQTL that affect canine hip dysplasia. In a recent study, Wang et al. [9] used reciprocal F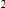 designs to identify the additive and dominant effects of iQTLs and their interactions with imprinting effects for hyperoxic acute lung injury survival time in mice. These authors also explore the transgenerational inheritance of iQTLs.

While epigenetic marks resulting in genomic imprinting can be generally stable in an organism's lifetime, they may undergo reprogramming, i.e., a faithful clearing of the epigenetic state established in the previous generation, in the new generation during gametogenesis and early embryogenesis [17], [18], . However, a growing body of evidence since the early 1980s indicates that genes may escape such reprogramming and, thus, inherit their imprinting effects into next generations [20], [21], [22], [23], [24], [25]. Two fundamental questions will naturally arise from this discovery: how common are imprinted genes of this type and how strong is the evidence for their existence in humans and other organisms? If epigenetic changes through imprinted genes can be inherited across generations, this would significantly alter the way we think about the inheritance of phenotype [26], [27]. Such transgenerational epigenetic inheritance, i.e., modifications of the chromosomes that pass to the next generation through gametes, may be related with health and diseases with a mechanism for transmitting environmental exposure information that alters gene expression in the next generation(s) [28]. The identification of imprinted loci displaying transgenerational epigenetic inheritance will be greatly helpful for addressing the two questions mentioned above, in a quest to elucidate the detailed genetic architecture of complex traits and diseases.

The motivation of this study is to develop a novel strategy for identifying imprinted genes for a quantitative trait and understanding the transgenerational changes of their effects with a three-generation family design by sampling multiple unrelated nuclear families, each composed of the grandfather, grandmother, father, mother, and grandchildren, from a natural population. This transgenerational design contains information about how alleles at different loci co-transmit during meiosis from one generation to next and, thus, has been widely used for genetic linkage analysis [29], [30]. By tracing the inheritance of alleles at a gene(s) from a paternal or maternal parent, this design allows the characterization of parent-of-origin of alleles and provides a powerful way to estimate genetic imprinting effects. Because only genotypes can be observed, we formulate a mixture model to specify allelic configurations in terms of parental origins of the alleles. The EM algorithm is implemented to estimate the effects of imprinted genes and their changes across generations. A testing procedure is proposed to study the pattern of transgenerational epigenetic inheritance. The statistical behavior of the model is examined through simulation studies.

## Results

Simulation studies were performed to examine the statistical behavior of the model. A three-generation design is simulated which include a certain number of first-generation families sorted into 9 mating types (as shown in Table S1) according to the genotype frequencies. Assume that the allele frequencies of a gene are 0.6 and 0.4 in a natural population at Hardy-Weinberg equilibrium. Our simulation will focus on the investigation of the impacts of different sampling strategies and heritabilities on parameter estimation and model power. For a given sample size, two sampling strategies are simulated, (1) a large family number and small family size, and (2) a small family number and large family size.

The first strategy samples 200 unrelated grandfathers and 200 unrelated grandmothers, who marry to form 200 the first-generation families. Each first-generation family is assumed to have one son who, as the father, form a second-generation family with the mother from the natural population. There is one child for each second-generation family. This allocation results in a total of 1000 subjects. All members in the design are typed for the gene, but only the fathers and offspring of the third generation are phenotyped for a normally distributed trait. The second strategy samples 50 unrelated grandfathers and 50 unrelated grandmothers. In each first-generation family, 3 sons are simulated, forming 150 second-generation families in which 4 children are assumed. This strategy also results in 1000 subjects.

Different genetic effects of the gene, additive, dominant, and imprinting, are simulated for the second- and third-generations using the designed shown in Tables S2 and S3. Two different heritability levels, 0.1 and 0.4, are simulated for each generation, from which variances are determined. Table 1 tabulates the estimates of population and quantitative genetic parameters from the three-generation design. As expected, allele frequency can be very well estimated. The model provides reasonable estimation accuracy and precision for all genetic parameters under different sampling strategies, even for a modest heritability level. Under both strategies, the model has great power (0.85 or higher) to test the significance of individual genetic effects, additive, dominant, and imprinting, expressed in different generations. The model is also powerful to detect differences of genetic effects between two consecutive generations. More interesting, the difference of imprinting effect between different generations, i.e., transgenerational inheritance of genetic imprinting, can be discerned with power 0.80 using our statistical design.

**Table 1:** The maximum likelihood estimates (MLEs) of additive (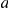), dominant (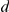), and imprinting effects (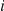) of a functional SNP on a complex trait in parental (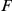) and offspring (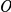) generations under two different strategies.

Genetic	True	Strategy 1		Strategy 2	
Parameter	Value	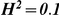	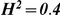	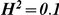	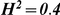
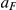	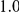	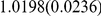	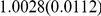	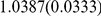	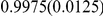
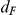	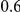	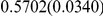	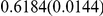	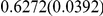	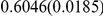
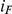	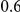	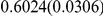	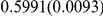	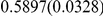	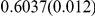
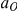	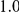	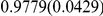	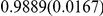	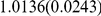	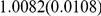
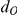	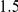	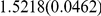	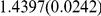	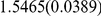	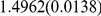
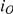	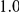	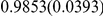	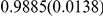	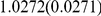	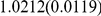

The esimates are the means of MLEs obtained from 200 simulation replicates, with standard errors given in parentheses.

One major aim of this study is to estimate the change of genetic effects over generation. Although our model has great power to detect the transgenerational change of genetic effects, its false positive rates should also be assessed. We conducted an additional simulation study to address this issue by simulating a SNP that has the same genetic effects between the two generations. The model detects a small proportion of simulation replicates (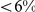) which displays transgenerational differences in all types of genetic effects including additive, dominant, and imprinting. This suggests that the model has a small type I error rate for detecting the transgenerational difference of overall genetic effects. We particularly tested the type I error rate for the transgenerational difference of genetic imprinting, which is reasonably small (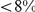).

The haplotype model is also examined through simulation studies. We simulated two SNPs with a recombination fraction of 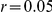 that are segregating in a human population. Of the four haplotypes, one is assumed to function as a risk haplotype. The remaining is collectively called the non-risk haplotype. The genetic values of composite diplotypes constituted by risk and non-risk haplotypes include the additive (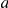), dominant (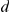), and imprinting (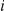) genetic effects. We assume that some of these effects are different, and the others are the same between the parental and offspring generations. Combinations of different heritabilities between the two generations are simulated.

Table 2 gives the results of simulation for different heritabilities and sample sizes (all subjects used). Overall, all parameters can be estimated reasonably well. As expected, the precision of parameter estimation increases with heritability and sample size. The additive genetic effects in both generations can well be estimated with a modest sample size (say 400) for a small heritability (0.1). More sample sizes (say 800) are needed to provide a good estimate for genetic imprinting effects for a small heritability. To well estimate dominant genetic effects, an even larger sample size (say 2000) is required for the same level of heritability.

**Table 2:** Simulation results for transgeneration imprinting effects comparisons.

				First Generation Parameters			Second Generation Parameters		
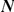	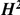		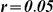	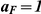	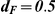	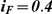	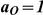	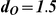	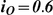
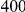	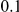	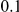	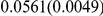	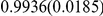	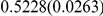	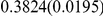	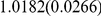	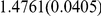	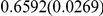
	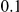	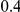	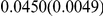	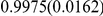	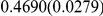	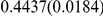	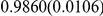	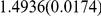	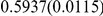
	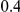	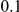	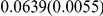	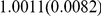	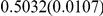	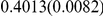	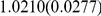	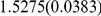	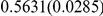
	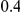	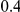	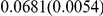	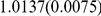	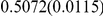	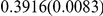	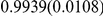	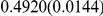	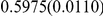
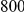	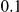	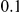	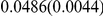	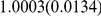	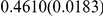	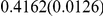	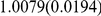	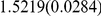	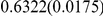
	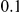	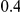	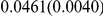	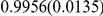	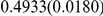	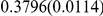	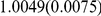	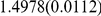	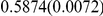
	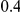	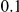	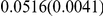	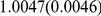	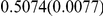	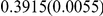	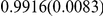	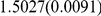	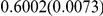
	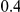	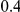	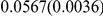	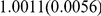	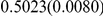	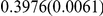	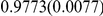	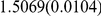	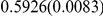
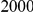	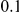	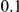	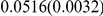	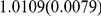	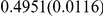	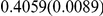	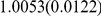	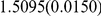	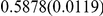
	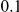	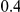	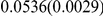	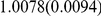	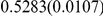	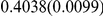	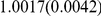	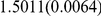	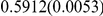
	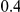	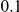	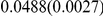	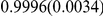	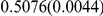	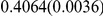	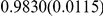	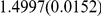	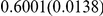
	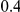	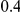	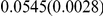	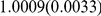	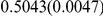	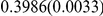	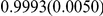	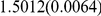	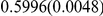

The genetic design scenarios are chosen as the combination of different heritabilities and sample sizes. They are: 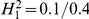, 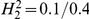, 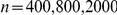.

## Discussion

The traditional view of quantitative trait expression analysis assumes that the maternally and paternally derived alleles of each gene are expressed simultaneously at a similar level. However, this view is violated by a growing body of evidence that alleles are expressed from only one of the two parental chromosomes [1], [2]. This so-called genetic imprinting or parent-of-origin effect has been thought to play a pivotal role in regulating the phenotypic variation of a complex trait [3], [4], [6], [8], [9], [12], [13], [14], [15]. With the discovery of more imprinting genes involved in trait control through molecular and bioinformatics approaches, we will be in a position to elucidate the genetic architecture of quantitative variation for various organisms including humans.

Recent evidence shows that epigenetic inheritance in humans may experience a transgenerational change. This would represent a significant shift in our current understanding of inheritance and disease aetiology. Despite the development of new technologies that are reducing the time and cost of genotyping by several orders of magnitude [31], [32], the understanding of the underlying genetic events will be challenging. In this article, we present a computational model for identifying the genomic imprinting effect of genes on quantitative phenotypes and transgenerational change of genomic imprinting using a multigenerational sampling design for human families. The model formulates a general framework for testing the difference of genetic effects between different generations. By including multiple SNPs, the model was extended to estimate genomic imprinting and its transgenerational change expressed at the haplotype level. Although several models have been developed to estimate genomic imprinting for binary disease traits [33], [34], our model is among the first for estimating genetic imprinting operational in regulating the variation of quantitative traits and is certainly the first of its kind that can discern the transgenerational change of genetic imprinting.

Although no real data were analyzed for the moment, this model presents a conceptual design by which new data can be collected according to the sampling strategy proposed and then analyzed by the computational algorithm derived. Based on computer simulation, the model should display convincing statistical properties in parameter estimation and test and can be applied to a practical data set. However, several issues need to be addressed when the model is attempted to solve broader genetic questions. First, the maternal effects that cause parent-of-origin effects of alleles may be confounded with imprinting effects [35], which should be separated by developing a proper design in order to better study the patterns of gene expression and evolutionary dynamics.

Second, this study assumes the unisex (sons) produced from the first-generation family. One can also assume daughters with no change of the model, allowing the test of genomic imprinting between mother and offspring. In fact, our model can involve both sexes so that in the second generation sex-specific genetic effects can be characterized. If the sexes in the third generation are considered, the model can be extended to study the transgenerational changes of gene-sex interactions. Third, it is possible that part of parental genotypes are missing in practice. To infer genomic imprinting using such data sets, a multi-hierachical mixture model can be derived to estimate the missing parental genotypes based on observed offspring genotypes. Fourth, although a basic premise of epigenetic processes was that, once established, these marks were maintained through rounds of mitotic cell division and stable for the life of the organism, several recent studies have shown that at some loci the epigenetic state can be altered by the environment [36]. The questions are how common are genes of this type and how strong is the evidence for their existence in humans? The development of our design and model will help to address these biological questions of fundamental importance in elucidating the genetic architecture of complex traits.

## Methods

### Sampling Strategies

Suppose there is a natural human population at Hardy–Weinberg equilibrium (HWE) from which a panel of three-generation families, each composed of the grandfather, grandmother, father, mother, and grandchildren, are sampled. Each member in a family is typed for single nucleotide polymorphisms (SNPs) from the human genome. Consider a quantitative trait affected by a SNP with two alleles *A* in a frequency of 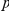 and *a* in a frequency of 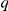, leading to three genotypes 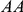, 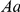, and 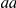 with the frequencies of 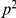, 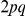, and 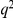, respectively. In the grandparent generation, these three genotypes are mating randomly to produce nine cross types (Table S1). Given a cross type, the genotypes of sons or daughters can be inferred. Here we first assume one sex (say son) in the second generation, although both sexes can be considered. The sons from a family serve as the father to mate with the females as the mother derived from a natural population, with genotypes, 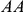, 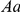, and 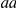, characterized by frequencies 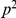, 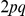, and 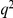, respectively. Each of such second-generation families produces a certain number of grandchildren. The genotype frequencies in the third generation are derived according to Mendel's first law.

According to this design, the grandfathers and grandmothers are founders whose parents are unknown. Alleles of sons from a first-generation family can be traced directly or indirectly, but the females used to generate the second-generation family are the founders with the unknown origin of alleles. For this reason, we will measure the phenotype for sons from the first-generation families and grandchildren from the second-generation families. This design will allow us to characterize imprinting effects of a gene in the second- and third-generations.

### Genetic Models

There are three genotypes, 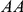, 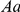, and 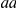, for a biallelic gene according to Mendelian segregation pattern. Considering the parent-of-origin of alleles, these genotypes are described by four configurations, 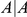 (coded as 2), 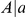 (coded as 1), 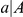 (coded as 1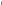), and 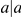 (coded as 0), where symbol 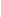 is used to separate the maternally- (left) and paternally-derived alleles (right). The genotypic values of the four configurations in two different generations are defined as follows:
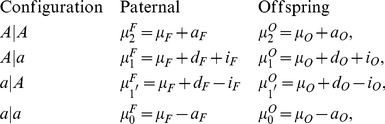
(1)where 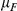 and 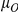 are the overall means of the paternal and offspring generations, 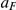, 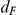, and 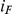 are the additive, dominant and imprinting genetic effects of the gene in the parental generation, and 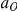, 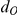, and 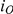 are the additive, dominant and imprinting genetic effects of the gene in the offspring generation.

The difference in the genetic architecture of a complex trait between two different generations is described as
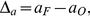
(2)

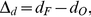
(3)

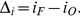
(4)By testing whether these differences are equal to zero jointly or individually, we can determine the transgenerational changes of the pattern of genetic control. If a significant imprinting effect is detected, we can test the type of genetic imprinting, i.e., parental or maternal dominance, by incorporating the imprinting models of Cheverud et al. [14].

### Estimation

The grandfather and grandmother in the first generation from a natural population constitutes 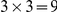 mating types for three genotypes. For the 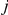
th first-generation mating type listed in Table S1 (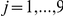), let 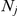 denote the family number of this mating type. Each first-generation family may have one or multiple sons who serve the father of the second generation. Those families in the second generation with the father derived from the 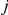
th first-generation mating type and the mother of a particular genotype from the natural population are summed together, denoted by 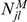, for mother genotype 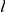 (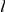 = 2 for 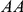, 1 for 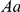, and 0 for 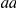). Thus, we have a total of 
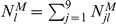
 second-generation mothers who carry genotype 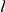.

It is not difficult to derive the maximum likelihood estimate of allele frequency from the three-generation family design as
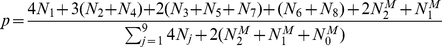


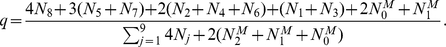



The male individuals from the first generation are typed for the marker, with four distinct configurations, 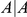 (2), 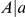 (1), 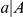 (1′), and 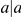 (0). Let 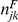 denote the cumulative number of male individuals (as the father for the second generation) bearing configuration 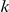 (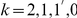) from 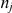 first-generation families. In the third generation, only genotypes rather than configurations can be observed. We use 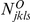 to denote the number of children who carry genotype 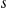 (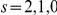) from a second-generation family with father 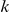 (from the 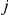
th first-generation mating type) and mother 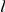 from a natural population. The phenotypic values measured are expressed as 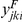 (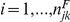) for the second-generation fathers and 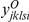 (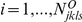) for the third-generation children. Both 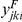 and 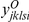 are assumed to follow a normal distribution with mean depending on genotypes and residual variances 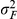 and 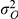, respectively.

Since offspring genotypes depend on parental genotypes, the log-likelihood of paternal and offspring parameters given marker (**M**) and phenotypic (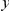) data from the three generations is decomposed into two components, one related to the paternal parameters and the second related to the offspring parameters given the paternal parameters, expressed as
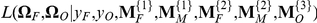


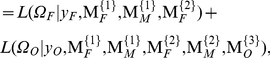
(5)where 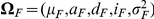 are the paternal parameters and 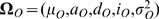 are the offspring parameters. Maximizing joint likelihood (5) is equivalent to maximizing its two likelihood components independently. The estimates of parameters 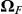 that maximize the first component can be obtained with the EM algorithm. In the E step, the posterior probability with which the double heterozygote father of the second generation from the 5th first-generation mating type in Table S1 has a particular configuration is calculated by
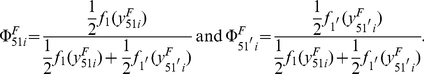
(6)In the M step, the genotypic values of configurations and variance are calculated by
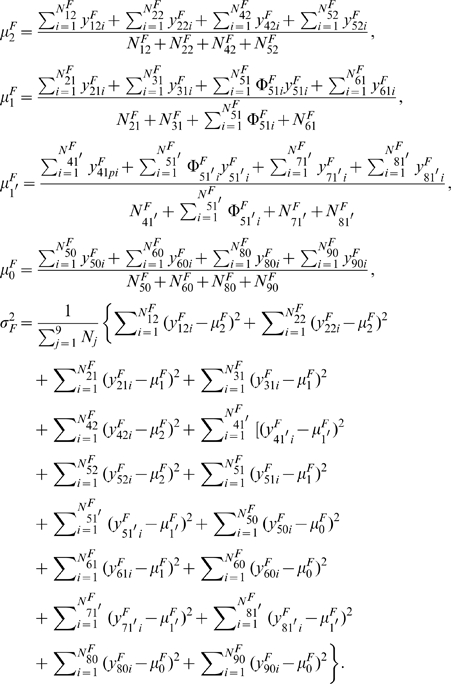
(7)


The EM algorithm can also be implemented to estimate genetic parameters 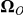 in the third generation that maximize the second component in (5). In the E step, the posterior probability with which the double heterozygote offspring of the third generation derived from the combination of two double heterozygote parents in the second generation has a particular configuration is calculated by
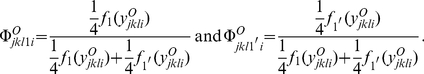
(8)In the M step, the genotypic values of configurations and variance are calculated by
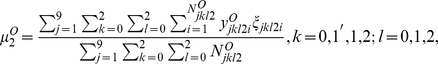


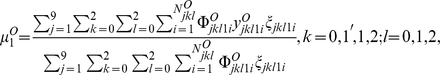


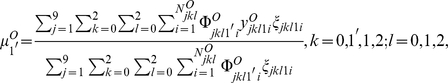


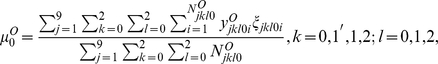


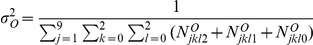


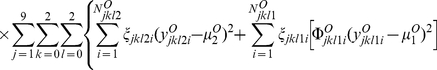


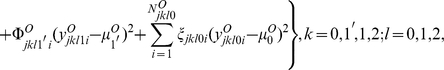
where 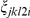, 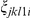, and 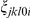 are the indicator variables that are defined as 1 if offspring 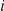 in the third generation from the combination of father 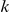 from the 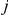
th first-generation mating type and mother 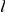 from the natural population has genotype 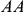, 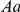, and 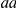, respectively, and 0 otherwise. The EM steps are iterated between equations (6) and (7) to obtain the MLEs of 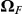 and between equations (8) and (9) to obtain the MLEs of 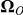.

### Hypothesis Tests

It is imperative to know whether there exists a significant association between a specific SNP and a complex trait and how a significant SNP triggers an additive, dominant, or imprinting effect on the trait. To test for the overall significant association of SNP genotype and trait phenotype, we generate the following hypotheses:
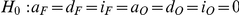


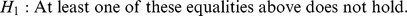
The log-likelihood ratio under the null and alternative hypotheses is calculated. Since the null hypothesis contains a nuisance parameter, allele frequency, this log-likelihood ratio test statistic may have an unclear distribution. For this reason, the critical threshold for claiming the existence of a significant SNP is determined from permutation tests [37]. If our interest is in testing whether there is an additive, dominant, or imprinting effect, the null hypothesis should be 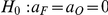, 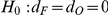, and 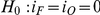, respectively. Because each of these null hypotheses is nested within its alternative, the log-likelihood ratio test statistic can be thought to asymptotically follow a 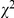-distribution for a large sample size.

The transgenerational changes of different genetic effects can also be tested. The null hypotheses used to test whether the additive, dominant, and imprinting effects display significant changes from one generation to next are expressed as 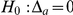, 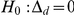, and 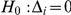, respectively. These null hypotheses can be considered singly or jointly, in order to better study the transgenerational changes of the genetic architecture of a trait.

### Haplotyping Model

Recent molecular surveys suggest that the human genome contains many discrete haplotype blocks that are sites of closely located SNPs [38], [39], [40]. Each block may have a few common haplotypes which account for a large proportion of chromosomal variation. Between adjacent blocks are there large regions, called hotspots, in which recombination events occur with high frequencies. Several algorithms have been developed to identify a minimal subset of SNPs, i.e., tagging SNPs, that can characterize the most common haplotypes [41]. The number and type of tagging SNPs within each haplotype block can be determined prior to association studies. In this section, we will derive a model for detecting the association between haplotypes constructed by alleles at a set of SNPs and complex traits.

For the simplicity of our description, consider two SNPs **A** (with two alleles *A* and *a*) and **B** (with two alleles *B* and *b*). They form four haplotypes 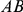, 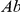, 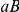, and 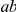, of which one that is distinct from the rest three is defined as a risk haplotype 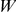 and all the others are defined as a non-risk haplotype 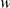 [42]. Risk and non-risk haplotypes from the maternal and paternal parents generate four composite diplotypes, 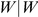, 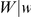, 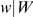, and 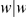, whose genotypic values are described by the additive (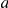), dominant (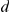), and imprinting genetic effects (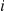). Cheng et al. [43] and Wang et al. [44] proposed a two- and three-SNP model for estimating and testing genetic imprinting effects in a natural population, respectively. Wu et al.'s procedure [45] allows the choice of an optimal number and combination of risk haplotypes within a multiallelic model framework. Here, we adopted Cheng et al.'s two-SNP model to estimate haplotype imprinting genetic effects and their transgenerational change.

In this example, four haplotypes 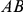, 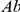, 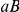, and 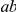 have frequencies denoted as 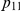, 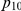, 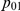, and 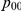, respectively. The two SNPs yield nine joint genotypes, *AABB* (coded as 1), *AABb* (coded as 2), …, *aabb* (coded as 9), which are actually observed. Each subject must bear one of these genotypes, and the parents in each family will be one of 9 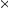 9 = 81 possible genotype by genotype combinations. If each parent for a combination is homozygous for both SNPs, their offspring will have one genotype. As long as one parent is heterozygous for one SNP, the offspring will have two or more genotypes. However, only when both SNPs are heterozygous for at least one parent, the genotype frequencies of offspring will be determined by the recombination fraction between the markers (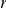). Tables S2 and S3 show the structure and frequencies of mother by father genotype combinations under random mating and their offspring genotype frequencies in the second and third generation, respectively. For a double heterozygote *AaBb*, its observed genotype may be derived from two possible diplotypes, 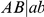 (with the relative proportion of 
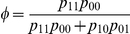
) or 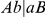 (with the relative proportion of 
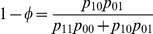
). Each of these two diplotypes produce four haplotypes 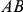, 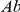, 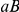, and 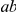, whose frequencies are expressed as


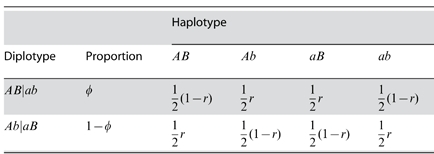


A similar likelihood (5) cane be formulated for haplotype models. A complicated EM algorithm is derived to estimate haplotype frequencies using the parental information. Let 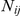 denote the observation of mating type between genotype 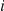 for one parent and genotype 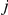 for the second parent. In the E step, calculate the proportion of a diplotype for a heterozygous genotype for a particular mating design by
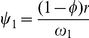


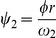


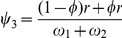


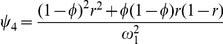


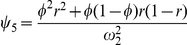


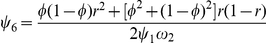


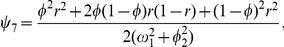
where 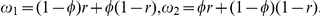


In the M step, estimate the haplotype frequencies and recombination fraction by
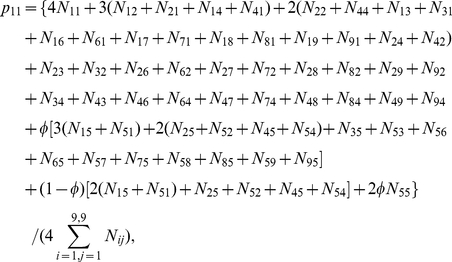


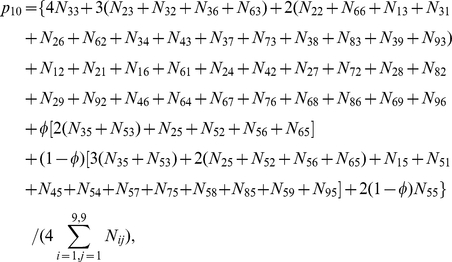


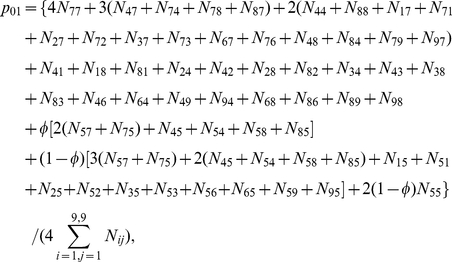


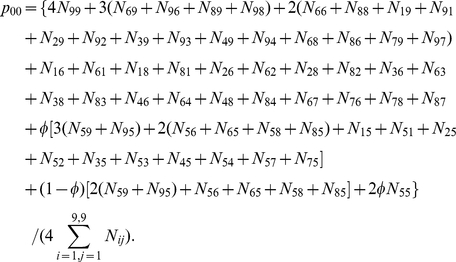


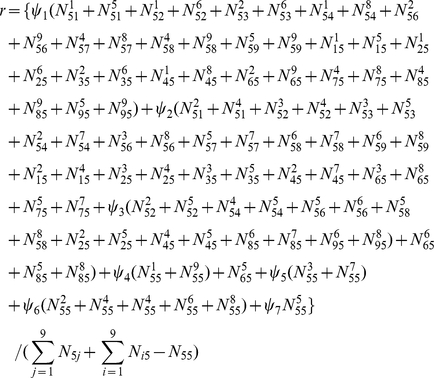



In the M step, the equations for estimating additive, dominant, imprinting effects expressed in paternal and offspring generations are also derived. The E and M steps are iterated until the estimates converge to a stable value. These stable values are the maximum likelihood estimates (MLEs) of parameters. The estimated haplotype frequencies and recombination fraction are embedded into a mixture model for estimating genotypic values and variances for different generations.

## Supporting Information

Table S1 A three-generation family design used to study transgenerational inheritance.
(PDF)

Table S2 A three-generation family design showing how to produce the second generation by mating different genotypes of grandfathers and grandmothers sampled from a natural population.
(PDF)

Table S3 A three-generation family design showing how to produce the second generation by mating different genotypes of grandfathers and grandmothers sampled from a natural population.
(PDF)

## References

[1] Reik W, Walter J (2001). Genomic imprinting: parental influence on the genome.. Nat Rev Genet.

[2] Wilkins JF, Haig D (2003). What good is genomic imprinting: The function of parent-specific gene expression.. Nat Rev Genet.

[3] Itier JM, Tremp G, Léonard JF, Multon MC (1998). Imprinted gene in postnatal growth role.. Nature.

[4] Li LL, Keverne EB, Aparicio SA, Ishino F (1999). Regulation of maternal behaviour and offpring growth by paternally expressed Peg3.. Science.

[5] Isles AR, Wilkinson LS (2000). Imprinted genes, cognition and behaviour.. Trend Cogn Sci.

[6] Constancia M, Kelsey G, Reik W (2004). Resourceful imprinting.. Nature.

[7] Wood AJ, Oakey RJ (2006). Genomic imprinting in mammals: Emerging themes and established theories.. PLoS Genet.

[8] Wilkinson LS, Davies W, Isles AR (2007). Genomic imprinting effects on brain development and function.. Nat Rev Neurosci.

[9] Wang CG, Wang Z, Luo JT, Li Q (2010). A model for transgenerational imprinting variation in complex traits.. PLoS ONE.

[10] Frost JM, Moore GE (2010). The importance of imprinting in the human placenta.. PLoS Genet.

[11] Sha K (2008). A mechanistic view of genomic imprinting.. Ann Rev Genom Hum Genet.

[12] De Koning DJ, Rattniek AP, Harlizius B, Arendonk JAM (2000). Genome-wide scan for body composition in pigs reveals important role of imprinting.. Proc Natl Acad Sci U S A.

[13] Liu T, Todhunter RJ, Wu S, Hou W (2007). A random model for mapping imprinted quantitative trait loci in a structured pedigree: An implication for mapping canine hip dysplasia.. Genomics.

[14] Cheverud JM, Hager R, Roseman C, Fawcett G (2008). Genomic imprinting effects on adult body composition in mice.. Proc Natl Acad Sci U S A.

[15] Wolf JB, Cheverud JM, Roseman C, Hager R (2008). Genome-wide analysis reveals a complex pattern of genomic imprinting in mice.. PLoS Genet.

[16] Li YC, Coelho CM, Liu T, Wu S (2007). A statistical strategy to estimate maternal-zygotic interactions and parent-of-origin effects of QTLs for seed development.. PLoS ONE.

[17] Morgan HD, Santos F, Green K, Dean W (2005). Epigenetic reprogramming in mammals.. Hum Mol Genet.

[18] Sasaki H, Matsui Y (2008). Epigenetic events in mammalian germ-cell development: reprogramming and beyond.. Nat Rev Genet.

[19] Tal O, Kisdi E, Jablonka E (2010). Epigenetic contribution to covariance between relatives.. Genetics.

[20] McGrath J, Solter D (1984). Inability of mouse blastomere nuclei transferred to enucleated zygotes to support development in vitro.. Science.

[21] Surani MA, Barton SC, Norris ML (1984). Development of reconstituted mouse eggs suggests imprinting of the genome during gametogenesis.. Nature.

[22] Morgan HD, Sutherland HG, Martin DI, Whitelaw E (1999). Epigenetic inheritance at the agouti locus in the mouse.. Nat Genet.

[23] Cropley JE, Suter CM, Beckman KB, Martin DI (2006). Germ-line epigenetic modification of the murine Avy allele by nutritional supplementation.. Proc Natl Acad Sci U S A.

[24] Skinner MK (2008). What is an epigenetic transgenerational phenotype? F3 or F2.. Reprod Toxic.

[25] Dolinoy DC, Weidman JR, Waterland RA, Jirtle RL (2006). Maternal genistein alters coat color and protects Avy mouse offspring from obesity by modifying the fetal epigenome.. Environ Health Perspect.

[26] Whitelaw NC, Whitelaw E (2008). Transgenerational epigenetic inheritance in health and disease.. Curr Opin Genet Dev.

[27] Youngson NA, Whitelaw E (2008). Transgenerational epigenetic effects.. Ann Rev Genom Hum Genet.

[28] Pembrey ME, Bygren LO, Kaati G, Edvinsson S (2006). Sex-specific, male-line transgenerational responses in humans.. Europ J Hum Genet.

[29] Wu RL, Ma CX, Casella G (2007). Statistical Genetics of Quantitative Traits: Linkage, Map, and QTLs.

[30] Li Q, Wu RL (2009). A multilocus model for constructing a linkage disequilibrium map in human populations.. Stat Appl Genet Mol Biol.

[31] Chan EY (2005). Advances in sequencing technology.. Mutant Res.

[32] Beckmann JS, Estivill X, Antonarakis SE (2007). Copy number variants and genetic traits: closer to the resolution of phenotypic to genotypic variability.. Nat Rev Genet.

[33] Weinberg CR, Wilcox AJ, Lie RT (1998). A log-linear approach to case-parent triad data: Assessing effects of disease genes that act directly or through maternal effects, and may be subject to parental imprinting.. Am J Hum Genet.

[34] Cordell HJ, Barratt BJ, Clayton DG (2004). Case/pseudocontrol analysis in genetic association studies: a unified framework for detection of genotype and haplotype associations, gene-gene and gene-environment interactions and parent-of-origin effects.. Genet Epid.

[35] Hager R, Cheverud JM, Wolf JB (2008). Maternal effects as the cause of parent-of-origin dependent effects that mimic genomic imprinting.. Genetics.

[36] Jirtle RL, Skinner MK (2007). Environmental epigenomics and disease susceptibility.. Nat Rev Genet.

[37] Churchill GA, Doerge RW (1994). Empirical threshold values for quantitative triat mapping.. Genetics.

[38] Dawson E, Abecasis GR, Bumpstead S, Chen Y (2002). A first-generation linkage disequilibrium map of human chromosome.. Nature.

[39] Gabriel SB, Schaffer SF, Nguyen H, Moore JM (2002). The structure of haplotype blocks in the human genome.. Science.

[40] Patil N, Berno AJ, Hinds DA, Barrett WA (2001). Blocks of limited haplotype diversity revealed by high-resolution scanning of human chromosome 21.. Science.

[41] Zhang K, Deng M, Chen T, Waterman MS, Sun F (2002). A dynamic programming algorithm for haplotype block partitioning.. Proc Natl Acad Sci U S A.

[42] Wu RL, Lin M (2008). Statistical and Computational Pharmacogenomics.

[43] Cheng Y, Berg A, Wu S, Li Y, Wu RL (2009). Computing genetic imprinting expressed by haplotypes.. Method Mol Biol.

[44] Wang CG, Cheng Y, Liu T, Li Q (2008). A computational model for sex-specific genetic architecture of complex traits in humans.. Mol Pain.

[45] Wu S, Yang J, Wang CG, Wu RL (2007). A general quantitative genetic model for haplotyping a complex trait in humans.. Curr Genom.

