# Supplementary material for: A Statistical Design for Testing Transgenerational Genomic Imprinting in Natural Human Populations
Source: PLoS One. 2011 Feb 25;6(2):e16858. doi: 10.1371/journal.pone.0016858 (PMC3045439; doi:10.1371/journal.pone.0016858)
Supplement: Table S1 — A three-generation family design used to study transgenerational inheritance. (PDF) [file pone.0016858.s001.pdf]

Table S1 A three-generation family design used to study transgenerational inheritance.

	First Generation			Second Generation			Third Generation			
Mating							AA	Aa		aa
Type	Father (Freq)		Mother (Freq)	Father (Freq)		Mother (Freq)	$A\|A$	$A\|a$	$a\|A$	$a\|a$
1	AA (p^2)	$\times$	AA (p^2)	$A\|A$ (1)	$\times$	AA (p^2)	1	0	0	0
						Aa ($2pq$)	$\frac{1}{2}$	$\frac{1}{2}$	0	0
						aa (q^2)	0	1	0	0
2	AA (p^2)	$\times$	Aa ($2pq$)	$A\|A$ ($\frac{1}{2}$)	$\times$	AA (p^2)	1	0	0	0
						Aa ($2pq$)	$\frac{1}{2}$	$\frac{1}{2}$	0	0
						aa (q^2)	0	1	0	0
				$A\|a$ ($\frac{1}{2}$)	$\times$	AA (p^2)	$\frac{1}{2}$	0	$\frac{1}{2}$	0
						Aa ($2pq$)	$\frac{1}{4}$	$\frac{1}{4}$	$\frac{1}{4}$	$\frac{1}{4}$
						aa (q^2)	0	$\frac{1}{2}$	0	$\frac{1}{2}$
3	AA (p^2)	$\times$	aa (q^2)	$A\|a$ (1)	$\times$	AA (p^2)	$\frac{1}{2}$	0	$\frac{1}{2}$	0
						Aa ($2pq$)	$\frac{1}{4}$	$\frac{1}{4}$	$\frac{1}{4}$	$\frac{1}{4}$
						aa (q^2)	0	$\frac{1}{2}$	0	$\frac{1}{2}$

Table S1: Continued

Mating	First Generation			Second Generation			Third Generation			
							AA	Aa		aa
Type	Father (Freq)		Mother (Freq)	Father (Freq)		Mother (Freq)	$A\|A$	$A\|a$	$a\|A$	$a\|a$
						AA (p^2)	1	0	0	0
4	Aa $(2pq)$	$\times$	AA (p^2)	$A\|A$ $(\frac{1}{2})$	$\times$	Aa $(2pq)$	$\frac{1}{2}$	$\frac{1}{2}$	0	0
						aa (q^2)	0	1	0	0
						AA (p^2)	$\frac{1}{2}$	0	$\frac{1}{2}$	0
				$a\|A$ $(\frac{1}{2})$	$\times$	Aa $(2pq)$	$\frac{1}{4}$	$\frac{1}{4}$	$\frac{1}{4}$	$\frac{1}{4}$
						aa (q^2)	0	$\frac{1}{2}$	0	$\frac{1}{2}$
						AA (p^2)	1	0	0	0
5	Aa $(2pq)$	$\times$	Aa $(2pq)$	$A\|A$ $(\frac{1}{4})$	$\times$	Aa $(2pq)$	$\frac{1}{2}$	$\frac{1}{2}$	0	0
						aa (q^2)	0	1	0	0
						AA (p^2)	$\frac{1}{2}$	0	$\frac{1}{2}$	0
				$A\|a$ $(\frac{1}{4})$	$\times$	Aa $(2pq)$	$\frac{1}{4}$	$\frac{1}{4}$	$\frac{1}{4}$	$\frac{1}{4}$
						aa (q^2)	0	$\frac{1}{2}$	0	$\frac{1}{2}$
						AA (p^2)	$\frac{1}{2}$	0	$\frac{1}{2}$	0
				$a\|A$ $(\frac{1}{4})$	$\times$	Aa $(2pq)$	$\frac{1}{4}$	$\frac{1}{4}$	$\frac{1}{4}$	$\frac{1}{4}$
						aa (q^2)	0	$\frac{1}{2}$	0	$\frac{1}{2}$
						AA (p^2)	0	0	1	0
				$a\|a$ $(\frac{1}{4})$	$\times$	Aa $(2pq)$	0	0	$\frac{1}{2}$	$\frac{1}{2}$
						aa (q^2)	0	0	0	1

Table S1: Continued

Mating Type	First Generation: Father (Freq)		First Generation: Mother (Freq)	Second Generation: Father (Freq)		Second Generation: Mother (Freq)	Third Generation: AA $A\|A$	Third Generation: Aa $A\|a$	Third Generation: Aa $a\|A$	Third Generation: aa $a\|a$
6	Aa $(2pq)$	$\times$	aa (q^2)	$A\|a$ $(\frac{1}{2})$	$\times$	AA (p^2)	$\frac{1}{2}$	0	$\frac{1}{2}$	0
						Aa $(2pq)$	$\frac{1}{4}$	$\frac{1}{4}$	$\frac{1}{4}$	$\frac{1}{4}$
						aa (q^2)	0	$\frac{1}{2}$	0	$\frac{1}{2}$
				$a\|a$ $(\frac{1}{2})$	$\times$	AA (p^2)	0	0	1	0
						Aa $(2pq)$	0	0	$\frac{1}{2}$	$\frac{1}{2}$
						aa (q^2)	0	0	0	1
7	aa (q^2)	$\times$	AA (p^2)	$a\|A$ (1)	$\times$	AA (p^2)	$\frac{1}{2}$	0	$\frac{1}{2}$	0
						Aa $(2pq)$	$\frac{1}{4}$	$\frac{1}{4}$	$\frac{1}{4}$	$\frac{1}{4}$
						aa (q^2)	0	$\frac{1}{2}$	0	$\frac{1}{2}$
8	aa (q^2)	$\times$	Aa $(2pq)$	$a\|A$ $(\frac{1}{2})$	$\times$	AA (p^2)	$\frac{1}{2}$	0	$\frac{1}{2}$	0
						Aa $(2pq)$	$\frac{1}{4}$	$\frac{1}{4}$	$\frac{1}{4}$	$\frac{1}{4}$
						aa (q^2)	0	$\frac{1}{2}$	0	$\frac{1}{2}$
				$a\|a$ $(\frac{1}{2})$	$\times$	AA (p^2)	0	0	1	0
						Aa $(2pq)$	0	0	$\frac{1}{2}$	$\frac{1}{2}$
						aa (q^2)	0	0	0	1
9	aa (q^2)	$\times$	aa (q^2)	$a\|a$ (1)	$\times$	AA (p^2)	0	0	1	0
						Aa $(2pq)$	0	0	$\frac{1}{2}$	$\frac{1}{2}$
						aa (q^2)	0	0	0	1
