# Supplementary material for: A Statistical Design for Testing Transgenerational Genomic Imprinting in Natural Human Populations
Source: PLoS One. 2011 Feb 25;6(2):e16858. doi: 10.1371/journal.pone.0016858 (PMC3045439; doi:10.1371/journal.pone.0016858)
Supplement: Table S3 — A three-generation family design showing how to produce the second generation by mating different genotypes of grandfathers and grandmothers sampled from a natural population. (PDF) [file pone.0016858.s003.pdf]

Table S2 A three-generation family design showing how to produce the third generation by mating different genotypes of grandfathers and grandmothers sampled from a natural population.

	First Generation		Second Generation															
			$AABB$	$AABb$		$AAbb$	$AaBB$		$AaBb$				$Aabb$		$aaBB$	$aaBb$		$aabb$
Mating	Father (Freq)	Mother (Freq)	$AB\|AB$	$AB\|Ab$	$Ab\|AB$	$Ab\|Ab$	$AB\|aB$	$aB\|AB$	$AB\|ab$	$Ab\|aB$	$aB\|Ab$	$ab\|AB$	$Ab\|ab$	$ab\|Ab$	$aB\|aB$	$aB\|ab$	$ab\|aB$	$ab\|ab$
Type			μ_2^O	μ_1^O	$\mu_{1'}^O$	μ_0^O	μ_1^O	$\mu_{1'}^O$	μ_1^O	μ_0^O	μ_0^O	$\mu_{1'}^O$	μ_0^O	μ_0^O	μ_0^O	μ_0^O	μ_0^O	μ_0^O
1	$AABB$ (p_{11}^2)	$AABB$ (p_{11}^2)	1															
2	$AABB$ (p_{11}^2)	$AABb$ ($2p_{11}p_{10}$)	$\frac{1}{2}$	$\frac{1}{2}$														
3	$AABB$ (p_{11}^2)	$AAbb$ (p_{10}^2)		1														
4	$AABB$ (p_{11}^2)	$AaBB$ ($2p_{11}p_{01}$)	$\frac{1}{2}$				$\frac{1}{2}$											
5	$AABB$ (p_{11}^2)	$AaBb$ { $AB\|ab$ ($2p_{11}p_{00}$)	$\frac{1}{2}\bar{r}_O$	$\frac{1}{2}r_O$			$\frac{1}{2}r_O$		$\frac{1}{2}\bar{r}_O$									
		$Ab\|aB$ ($2p_{10}p_{01}$)	$\frac{1}{2}r_O$	$\frac{1}{2}\bar{r}_O$			$\frac{1}{2}\bar{r}_O$		$\frac{1}{2}r_O$									
6	$AABB$ (p_{11}^2)	$Aabb$ ($2p_{10}p_{00}$)		$\frac{1}{2}$					$\frac{1}{2}$									
7	$AABB$ (p_{11}^2)	$aaBB$ (p_{01}^2)					1											
8	$AABB$ (p_{11}^2)	$aaBb$ ($2p_{01}p_{00}$)					$\frac{1}{2}$		$\frac{1}{2}$									
9	$AABB$ (p_{11}^2)	$aabb$ (p_{00}^2)							1									
10	$AABb$ ($2p_{11}p_{10}$)	$AABB$ (p_{11}^2)	$\frac{1}{2}$		$\frac{1}{2}$													
11	$AABb$ ($2p_{11}p_{10}$)	$AABb$ ($2p_{11}p_{10}$)	$\frac{1}{4}$	$\frac{1}{4}$	$\frac{1}{4}$	$\frac{1}{4}$												
12	$AABb$ ($2p_{11}p_{10}$)	$AAbb$ (p_{10}^2)		$\frac{1}{2}$		$\frac{1}{2}$												
13	$AABb$ ($2p_{11}p_{10}$)	$AaBB$ ($2p_{11}p_{01}$)	$\frac{1}{4}$		$\frac{1}{4}$		$\frac{1}{4}$			$\frac{1}{4}$								
14	$AABb$ ($2p_{11}p_{10}$)	$AaBb$ { $AB\|ab$ ($2p_{11}p_{00}$)	$\frac{1}{4}\bar{r}_O$	$\frac{1}{4}r_O$	$\frac{1}{4}\bar{r}_O$	$\frac{1}{4}r_O$	$\frac{1}{4}r_O$		$\frac{1}{4}\bar{r}_O$	$\frac{1}{4}r_O$			$\frac{1}{4}\bar{r}_O$					
		$Ab\|aB$ ($2p_{10}p_{01}$)	$\frac{1}{4}r_O$	$\frac{1}{4}\bar{r}_O$	$\frac{1}{4}r_O$	$\frac{1}{4}\bar{r}_O$	$\frac{1}{4}\bar{r}_O$		$\frac{1}{4}r_O$	$\frac{1}{4}\bar{r}_O$			$\frac{1}{4}r_O$					
15	$AABb$ ($2p_{11}p_{10}$)	$Aabb$ ($2p_{10}p_{00}$)		$\frac{1}{4}$		$\frac{1}{4}$			$\frac{1}{4}$				$\frac{1}{4}$					
16	$AABb$ ($2p_{11}p_{10}$)	$aaBB$ (p_{01}^2)					$\frac{1}{2}$			$\frac{1}{2}$								
17	$AABb$ ($2p_{11}p_{10}$)	$aaBb$ ($2p_{01}p_{00}$)					$\frac{1}{4}$		$\frac{1}{4}$	$\frac{1}{4}$			$\frac{1}{4}$					
18	$AABb$ ($2p_{11}p_{10}$)	$aabb$ (p_{00}^2)							$\frac{1}{2}$				$\frac{1}{2}$					
19	$AAbb$ (p_{10}^2)	$AABB$ (p_{11}^2)			1													
20	$AAbb$ (p_{10}^2)	$AABb$ ($2p_{11}p_{10}$)			$\frac{1}{2}$	$\frac{1}{2}$												
21	$AAbb$ (p_{10}^2)	$AAbb$ (p_{10}^2)				1												
22	$AAbb$ (p_{10}^2)	$AaBB$ ($2p_{11}p_{01}$)			$\frac{1}{2}$					$\frac{1}{2}$								
23	$AAbb$ (p_{10}^2)	$AaBb$ { $AB\|ab$ ($2p_{11}p_{00}$)			$\frac{1}{2}\bar{r}_O$	$\frac{1}{2}r_O$				$\frac{1}{2}r_O$			$\frac{1}{2}\bar{r}_O$					
		$Ab\|aB$ ($2p_{10}p_{01}$)			$\frac{1}{2}r_O$	$\frac{1}{2}\bar{r}_O$				$\frac{1}{2}\bar{r}_O$			$\frac{1}{2}r_O$					

Table S3: Continued

	First Generation		Second Generation															
			$AABB$	$AABb$		$AAbb$	$AaBB$		$AaBb$				$Aabb$		$aaBB$	$aaBb$		$aabb$
Mating	Father (Freq)	Mother (Freq)	$AB\|AB$	$AB\|Ab$	$Ab\|AB$	$Ab\|Ab$	$AB\|aB$	$aB\|AB$	$AB\|ab$	$Ab\|aB$	$aB\|Ab$	$ab\|AB$	$Ab\|ab$	$ab\|Ab$	$aB\|aB$	$aB\|ab$	$ab\|aB$	$ab\|ab$
Type			μ_2^O	μ_1^O	$\mu_{1'}^O$	μ_0^O	μ_1^O	$\mu_{1'}^O$	μ_1^O	μ_0^O	μ_0^O	$\mu_{1'}^O$	μ_0^O	μ_0^O	μ_0^O	μ_0^O	μ_0^O	μ_0^O
24	$AAbb$ (p_{10}^2)	$Aabb$ ($2p_{10}p_{00}$)				$\frac{1}{2}$							$\frac{1}{2}$					
25	$AAbb$ (p_{10}^2)	$aaBB$ (p_{01}^2)							1									
26	$AAbb$ (p_{10}^2)	$aaBb$ ($2p_{01}p_{00}$)								$\frac{1}{2}$			$\frac{1}{2}$					
27	$AAbb$ (p_{10}^2)	$aabb$ (p_{00}^2)											1					
28	$AaBB$ ($2p_{11}p_{01}$)	$AABB$ (p_{11}^2)	$\frac{1}{2}$					$\frac{1}{2}$										
29	$AaBB$ ($2p_{11}p_{01}$)	$AABb$ ($2p_{11}p_{10}$)	$\frac{1}{4}$	$\frac{1}{4}$				$\frac{1}{4}$			$\frac{1}{4}$							
30	$AaBB$ ($2p_{11}p_{01}$)	$AAbb$ (p_{10}^2)		$\frac{1}{2}$							$\frac{1}{2}$							
31	$AaBB$ ($2p_{11}p_{01}$)	$AaBB$ ($2p_{11}p_{01}$)	$\frac{1}{4}$				$\frac{1}{4}$	$\frac{1}{4}$							$\frac{1}{4}$			
32	$AaBB$ ($2p_{11}p_{01}$)	$AaBb$ $AB\|ab$ ($2p_{11}p_{00}$)	$\frac{1}{4}\bar{r}_O$	$\frac{1}{4}r_O$			$\frac{1}{4}r_O$	$\frac{1}{4}\bar{r}_O$	$\frac{1}{4}\bar{r}_O$		$\frac{1}{4}r_O$				$\frac{1}{4}r_O$	$\frac{1}{4}\bar{r}_O$		
		$Ab\|aB$ ($2p_{10}p_{01}$)	$\frac{1}{4}r_O$	$\frac{1}{4}\bar{r}_O$			$\frac{1}{4}\bar{r}_O$	$\frac{1}{4}r_O$	$\frac{1}{4}r_O$		$\frac{1}{4}\bar{r}_O$				$\frac{1}{4}\bar{r}_O$	$\frac{1}{4}r_O$		
33	$AaBB$ ($2p_{11}p_{01}$)	$Aabb$ ($2p_{10}p_{00}$)		$\frac{1}{4}$					$\frac{1}{4}$		$\frac{1}{4}$					$\frac{1}{4}$		
34	$AaBB$ ($2p_{11}p_{01}$)	$aaBB$ (p_{01}^2)					$\frac{1}{2}$								$\frac{1}{2}$			
35	$AaBB$ ($2p_{11}p_{01}$)	$aaBb$ ($2p_{01}p_{00}$)					$\frac{1}{4}$	$\frac{1}{4}$						$\frac{1}{4}$	$\frac{1}{4}$			
36	$AaBB$ ($2p_{11}p_{01}$)	$aabb$ (p_{00}^2)							$\frac{1}{2}$		$\frac{1}{2}$							
37	$AaBb$ $AB\|ab$ ($2p_{11}p_{00}$)	$AABB$ (p_{11}^2)	$\frac{1}{2}\bar{r}_O$		$\frac{1}{2}r_O$			$\frac{1}{2}r_O$								$\frac{1}{2}\bar{r}_O$		
	$Ab\|aB$ ($2p_{10}p_{01}$)		$\frac{1}{2}r_O$		$\frac{1}{2}\bar{r}_O$			$\frac{1}{2}\bar{r}_O$								$\frac{1}{2}r_O$		
38	$AaBb$ $AB\|ab$ ($2p_{11}p_{00}$)	$AABb$ ($2p_{11}p_{10}$)	$\frac{1}{4}\bar{r}_O$	$\frac{1}{4}\bar{r}_O$	$\frac{1}{4}r_O$	$\frac{1}{4}r_O$		$\frac{1}{4}r_O$			$\frac{1}{4}r_O$	$\frac{1}{4}\bar{r}_O$		$\frac{1}{4}\bar{r}_O$				
	$Ab\|aB$ ($2p_{10}p_{01}$)		$\frac{1}{4}r_O$	$\frac{1}{4}r_O$	$\frac{1}{4}\bar{r}_O$	$\frac{1}{4}\bar{r}_O$		$\frac{1}{4}\bar{r}_O$			$\frac{1}{4}\bar{r}_O$	$\frac{1}{4}r_O$		$\frac{1}{4}r_O$				
39	$AaBb$ $AB\|ab$ ($2p_{11}p_{00}$)	$AAbb$ (p_{10}^2)		$\frac{1}{2}\bar{r}_O$		$\frac{1}{2}r_O$					$\frac{1}{2}r_O$			$\frac{1}{2}\bar{r}_O$				
	$Ab\|aB$ ($2p_{10}p_{01}$)			$\frac{1}{2}r_O$		$\frac{1}{2}\bar{r}_O$					$\frac{1}{2}\bar{r}_O$			$\frac{1}{2}r_O$				
40	$AaBb$ $AB\|ab$ ($2p_{11}p_{00}$)	$AaBB$ ($2p_{11}p_{01}$)	$\frac{1}{4}\bar{r}_O$		$\frac{1}{4}r_O$		$\frac{1}{4}\bar{r}_O$	$\frac{1}{4}r_O$		$\frac{1}{4}r_O$		$\frac{1}{4}\bar{r}_O$			$\frac{1}{4}r_O$		$\frac{1}{4}\bar{r}_O$	
	$Ab\|aB$ ($2p_{10}p_{01}$)		$\frac{1}{4}r_O$		$\frac{1}{4}\bar{r}_O$		$\frac{1}{4}r_O$	$\frac{1}{4}\bar{r}_O$		$\frac{1}{4}\bar{r}_O$		$\frac{1}{4}r_O$			$\frac{1}{4}\bar{r}_O$		$\frac{1}{4}r_O$	
41	$AaBb$ $AB\|ab$ ($2p_{11}p_{00}$)	$AaBb$ $AB\|ab$ ($2p_{11}p_{00}$)	$\frac{1}{4}\bar{r}_O^2$	$\frac{1}{4}\bar{r}_O r$	$\frac{1}{4}r_O\bar{r}_O$	$\frac{1}{4}r_O^2$	$\frac{1}{4}\bar{r}_O r$	$\frac{1}{4}r_O\bar{r}_O$	$\frac{1}{4}\bar{r}_O^2$	$\frac{1}{4}r_O^2$	$\frac{1}{4}r_O^2$	$\frac{1}{4}\bar{r}_O r$	$\frac{1}{4}r_O\bar{r}_O$	$\frac{1}{4}\bar{r}_O r$	$\frac{1}{4}r_O^2$	$\frac{1}{4}r_O\bar{r}_O$	$\frac{1}{4}\bar{r}_O r$	$\frac{1}{4}\bar{r}_O^2$
	$Ab\|aB$ ($2p_{10}p_{01}$)	$Ab\|aB$ ($2p_{10}p_{01}$)	$\frac{1}{4}r_O^2$	$\frac{1}{4}r_O\bar{r}_O$	$\frac{1}{4}\bar{r}_O r$	$\frac{1}{4}\bar{r}_O^2$	$\frac{1}{4}r_O\bar{r}_O$	$\frac{1}{4}\bar{r}_O r$	$\frac{1}{4}r_O^2$	$\frac{1}{4}\bar{r}_O^2$	$\frac{1}{4}\bar{r}_O^2$	$\frac{1}{4}r_O\bar{r}_O$	$\frac{1}{4}\bar{r}_O r$	$\frac{1}{4}r_O\bar{r}_O$	$\frac{1}{4}\bar{r}_O^2$	$\frac{1}{4}\bar{r}_O r$	$\frac{1}{4}r_O\bar{r}_O$	$\frac{1}{4}r_O^2$
42	$AaBb$ $AB\|ab$ ($2p_{11}p_{00}$)	$Aabb$ ($2p_{10}p_{00}$)		$\frac{1}{4}\bar{r}_O$		$\frac{1}{4}r_O$			$\frac{1}{4}\bar{r}_O$		$\frac{1}{4}r_O$		$\frac{1}{4}r_O$	$\frac{1}{4}\bar{r}_O$		$\frac{1}{4}r_O$		$\frac{1}{4}\bar{r}_O$
	$Ab\|aB$ ($2p_{10}p_{01}$)			$\frac{1}{4}r_O$		$\frac{1}{4}\bar{r}_O$			$\frac{1}{4}r_O$		$\frac{1}{4}\bar{r}_O$		$\frac{1}{4}\bar{r}_O$	$\frac{1}{4}r_O$		$\frac{1}{4}\bar{r}_O$		$\frac{1}{4}r_O$
43	$AaBb$ $AB\|ab$ ($2p_{11}p_{00}$)	$aaBB$ (p_{01}^2)					$\frac{1}{2}\bar{r}_O$			$\frac{1}{2}r_O$					$\frac{1}{2}r_O$		$\frac{1}{2}\bar{r}_O$	
	$Ab\|aB$ ($2p_{10}p_{01}$)						$\frac{1}{2}r_O$			$\frac{1}{2}\bar{r}_O$					$\frac{1}{2}\bar{r}_O$		$\frac{1}{2}r_O$	
44	$AaBb$ $AB\|ab$ ($2p_{11}p_{00}$)	$aaBb$ ($2p_{01}p_{00}$)					$\frac{1}{4}\bar{r}_O$		$\frac{1}{4}\bar{r}_O$	$\frac{1}{4}r_O$			$\frac{1}{4}r_O$		$\frac{1}{4}r_O$	$\frac{1}{4}r_O$	$\frac{1}{4}\bar{r}_O$	$\frac{1}{4}\bar{r}_O$
	$Ab\|aB$ ($2p_{10}p_{01}$)						$\frac{1}{4}r_O$		$\frac{1}{4}r_O$	$\frac{1}{4}\bar{r}_O$			$\frac{1}{4}\bar{r}_O$		$\frac{1}{4}\bar{r}_O$	$\frac{1}{4}\bar{r}_O$	$\frac{1}{4}r_O$	$\frac{1}{4}r_O$

Table S3: Continued

Mating Type	First Generation: Father (Freq)	First Generation: Mother (Freq)	Second Generation: $AABB$ $AB\mid AB$ μ_2^O	$AABb$ $AB\mid Ab$ μ_1^O	$AABb$ $Ab\mid AB$ $\mu_{1'}^O$	$AAbb$ $Ab\mid Ab$ μ_0^O	$AaBB$ $AB\mid aB$ μ_1^O	$AaBB$ $aB\mid AB$ $\mu_{1'}^O$	$AaBb$ $AB\mid ab$ μ_1^O	$AaBb$ $Ab\mid aB$ μ_0^O	$AaBb$ $aB\mid Ab$ μ_0^O	$AaBb$ $ab\mid AB$ $\mu_{1'}^O$	$Aabb$ $Ab\mid ab$ μ_0^O	$Aabb$ $ab\mid Ab$ μ_0^O	$aaBB$ $aB\mid aB$ μ_0^O	$aaBb$ $aB\mid ab$ μ_0^O	$aaBb$ $ab\mid aB$ μ_0^O	$aabb$ $ab\mid ab$ μ_0^O
45	$AaBb$ $AB\mid ab$ $(2p_{11}p_{00})$	$aabb$ (p_{00}^2)							$\frac{1}{2}\bar{r}_O$				$\frac{1}{2}r_O$			$\frac{1}{2}r_O$		$\frac{1}{2}\bar{r}_O$
	$AaBb$ $Ab\mid aB$ $(2p_{10}p_{01})$								$\frac{1}{2}r_O$				$\frac{1}{2}\bar{r}_O$			$\frac{1}{2}\bar{r}_O$		$\frac{1}{2}r_O$
46	$Aabb$ $(2p_{10}p_{00})$	$AABB$ (p_{11}^2)			$\frac{1}{2}$							$\frac{1}{2}$						
47	$Aabb$ $(2p_{10}p_{00})$	$AABb$ $(2p_{11}p_{10})$			$\frac{1}{4}$	$\frac{1}{4}$						$\frac{1}{4}$		$\frac{1}{4}$				
48	$Aabb$ $(2p_{10}p_{00})$	$AAbb$ (p_{10}^2)				$\frac{1}{2}$								$\frac{1}{2}$				
49	$Aabb$ $(2p_{10}p_{00})$	$AaBB$ $(2p_{11}p_{01})$			$\frac{1}{4}$					$\frac{1}{4}$		$\frac{1}{4}$					$\frac{1}{4}$	
50	$Aabb$ $(2p_{10}p_{00})$	$AaBb$ $AB\mid ab$ $(2p_{11}p_{00})$			$\frac{1}{4}\bar{r}_O$	$\frac{1}{4}r_O$				$\frac{1}{4}r_O$		$\frac{1}{4}\bar{r}_O$	$\frac{1}{4}\bar{r}_O$	$\frac{1}{4}r_O$			$\frac{1}{4}r_O$	$\frac{1}{4}\bar{r}_O$
		$AaBb$ $Ab\mid aB$ $(2p_{10}p_{01})$			$\frac{1}{4}r_O$	$\frac{1}{4}\bar{r}_O$				$\frac{1}{4}\bar{r}_O$		$\frac{1}{4}r_O$	$\frac{1}{4}r_O$	$\frac{1}{4}\bar{r}_O$			$\frac{1}{4}\bar{r}_O$	$\frac{1}{4}r_O$
51	$Aabb$ $(2p_{10}p_{00})$	$Aabb$ $(2p_{10}p_{00})$				$\frac{1}{4}$							$\frac{1}{4}$	$\frac{1}{4}$				$\frac{1}{4}$
52	$Aabb$ $(2p_{10}p_{00})$	$aaBB$ (p_{01}^2)								$\frac{1}{2}$							$\frac{1}{2}$	
53	$Aabb$ $(2p_{10}p_{00})$	$aaBb$ $(2p_{01}p_{00})$								$\frac{1}{4}$			$\frac{1}{4}$				$\frac{1}{4}$	$\frac{1}{4}$
54	$Aabb$ $(2p_{10}p_{00})$	$aabb$ (p_{00}^2)											$\frac{1}{2}$					$\frac{1}{2}$
55	$aaBB$ (p_{01}^2)	$AABB$ (p_{11}^2)						1										
56	$aaBB$ (p_{01}^2)	$AABb$ $(2p_{11}p_{10})$						$\frac{1}{2}$			$\frac{1}{2}$							
57	$aaBB$ (p_{01}^2)	$AAbb$ (p_{10}^2)									1							
58	$aaBB$ (p_{01}^2)	$AaBB$ $(2p_{11}p_{01})$						$\frac{1}{2}$							$\frac{1}{2}$			
59	$aaBB$ (p_{01}^2)	$AaBb$ $AB\mid ab$ $(2p_{11}p_{00})$						$\frac{1}{2}\bar{r}_O$			$\frac{1}{2}r_O$				$\frac{1}{2}r_O$	$\frac{1}{2}\bar{r}_O$		
		$AaBb$ $Ab\mid aB$ $(2p_{10}p_{01})$						$\frac{1}{2}r_O$			$\frac{1}{2}\bar{r}_O$				$\frac{1}{2}\bar{r}_O$	$\frac{1}{2}r_O$		
60	$aaBB$ (p_{01}^2)	$Aabb$ $(2p_{10}p_{00})$									$\frac{1}{2}$					$\frac{1}{2}$		
61	$aaBB$ (p_{01}^2)	$aaBB$ (p_{01}^2)													1			
62	$aaBB$ (p_{01}^2)	$aaBb$ $(2p_{01}p_{00})$													$\frac{1}{2}$	$\frac{1}{2}$		
63	$aaBB$ (p_{01}^2)	$aabb$ (p_{00}^2)														1		
64	$aaBb$ $(2p_{01}p_{00})$	$AABB$ (p_{11}^2)						$\frac{1}{2}$				$\frac{1}{2}$						
65	$aaBb$ $(2p_{01}p_{00})$	$AABb$ $(2p_{11}p_{10})$						$\frac{1}{4}$			$\frac{1}{4}$	$\frac{1}{4}$		$\frac{1}{4}$				
66	$aaBb$ $(2p_{01}p_{00})$	$AAbb$ (p_{10}^2)									$\frac{1}{2}$			$\frac{1}{2}$				
67	$aaBb$ $(2p_{01}p_{00})$	$AaBB$ $(2p_{11}p_{01})$						$\frac{1}{4}$				$\frac{1}{4}$			$\frac{1}{4}$		$\frac{1}{4}$	

Table S3: Continued

Mating Type	First Generation			Second Generation															
				$AABB$	$AABb$		$AAbb$	$AaBB$		$AaBb$						$Aabb$		$aaBB$	$aaBb$		$aabb$
	Father (Freq)	Mother (Freq)		$AB\|AB$	$AB\|Ab$	$Ab\|AB$	$Ab\|Ab$	$AB\|aB$	$aB\|AB$	$AB\|ab$	$Ab\|aB$	$aB\|Ab$	$ab\|AB$	$Ab\|ab$	$ab\|Ab$	$aB\|aB$	$aB\|ab$	$ab\|aB$	$ab\|ab$
Type				μ_2^O	μ_1^O	$\mu_{1'}^O$	μ_0^O	μ_1^O	$\mu_{1'}^O$	μ_1^O	μ_0^O	μ_0^O	$\mu_{1'}^O$	μ_0^O	μ_0^O	μ_0^O	μ_0^O	μ_0^O	μ_0^O
68	$aaBb$ $(2p_{01}p_{00})$	$AaBb$	$AB\|ab$ $(2p_{11}p_{00})$						$\frac{1}{4}\bar{r}_O$			$\frac{1}{4}r_O$	$\frac{1}{4}\bar{r}_O$		$\frac{1}{4}r_O$	$\frac{1}{4}r_O$	$\frac{1}{4}\bar{r}_O$	$\frac{1}{4}r_O$	$\frac{1}{4}\bar{r}_O$
			$Ab\|aB$ $(2p_{10}p_{01})$						$\frac{1}{4}r_O$			$\frac{1}{4}\bar{r}_O$	$\frac{1}{4}r_O$		$\frac{1}{4}\bar{r}_O$	$\frac{1}{4}\bar{r}_O$	$\frac{1}{4}r_O$	$\frac{1}{4}\bar{r}_O$	$\frac{1}{4}r_O$
69	$aaBb$ $(2p_{01}p_{00})$	$Aabb$ $(2p_{10}p_{00})$										$\frac{1}{4}$			$\frac{1}{4}$		$\frac{1}{4}$		$\frac{1}{4}$
70	$aaBb$ $(2p_{01}p_{00})$	$aaBB$ (p_{01}^2)														$\frac{1}{2}$		$\frac{1}{2}$	
71	$aaBb$ $(2p_{01}p_{00})$	$aaBb$ $(2p_{01}p_{00})$														$\frac{1}{4}$	$\frac{1}{4}$	$\frac{1}{4}$	$\frac{1}{4}$
72	$aaBb$ $(2p_{01}p_{00})$	$aabb$ (p_{00}^2)														$\frac{1}{2}$		$\frac{1}{2}$	
73	$aabb$ (p_{00}^2)	$AABB$ (p_{11}^2)											1						
74	$aabb$ (p_{00}^2)	$AABb$ $(2p_{11}p_{10})$											$\frac{1}{2}$		$\frac{1}{2}$				
75	$aabb$ (p_{00}^2)	$AAbb$ (p_{10}^2)													1				
76	$aabb$ (p_{00}^2)	$AaBB$ $(2p_{11}p_{01})$											$\frac{1}{2}$					$\frac{1}{2}$	
77	$aabb$ (p_{00}^2)	$AaBb$	$AB\|ab$ $(2p_{11}p_{00})$										$\frac{1}{2}\bar{r}_O$		$\frac{1}{2}r_O$			$\frac{1}{2}r_O$	$\frac{1}{2}\bar{r}_O$
			$Ab\|aB$ $(2p_{10}p_{01})$										$\frac{1}{2}r_O$		$\frac{1}{2}\bar{r}_O$			$\frac{1}{2}\bar{r}_O$	$\frac{1}{2}r_O$
78	$aabb$ (p_{00}^2)	$Aabb$ $(2p_{10}p_{00})$													$\frac{1}{2}$				$\frac{1}{2}$
79	$aabb$ (p_{00}^2)	$aaBB$ (p_{01}^2)																1	
80	$aabb$ (p_{00}^2)	$aaBb$ $(2p_{01}p_{00})$																$\frac{1}{2}$	$\frac{1}{2}$
81	$aabb$ (p_{00}^2)	$aabb$ (p_{00}^2)																	1
